# Applicability of ESPGHAN Biopsy-Free Guidelines for Celiac Disease Diagnosis: Insights from Türkiye

**DOI:** 10.5152/tjg.2025.24718

**Published:** 2025-03-19

**Authors:** Zeren Barış, Müberra Canbaz, Beyzanur Yılmaz, Neslihan Üstün, Yusuf Aydemir

**Affiliations:** 1 Department of Pediatric Gastroenterology, Eskişehir Osmangazi University Faculty of Medicine, Eskişehir, Türkiye; 2 Department of Pediatrics, Eskişehir Osmangazi University Faculty of Medicine, Eskişehir, Türkiye

**Keywords:** Adult, Celiac disease, non-biopsy diagnosis, pediatric

## Abstract

**Background/Aims:**

The biopsy-free diagnostic approach for celiac disease (CD) in children, recommended by ESPGHAN guidelines, is not widely implemented in pediatric gastroenterology centers across Türkiye. This study aimed to retrospectively evaluate patients who met ESPGHAN biopsy-free criteria but were nonetheless diagnosed through biopsy.

**Materials and Methods:**

Of 180 pediatric patients diagnosed with CD in the authors’ department over 5 years, 79 (43.8%) met the ESPGHAN biopsy-free criteria. All patients underwent routine biopsies of the duodenum, bulb, antrum, corpus, and esophagus at diagnosis. Clinical presentations, celiac serology, and endoscopic and histopathological findings were retrospectively analyzed.

**Results:**

The mean age at diagnosis was 7.48 ± 4 years (range, 2-17; M/F: 38/41). Presenting symptoms included growth failure (32.9%), abdominal pain (22.8%), constipation (8.9%), diarrhea (7.6%), anemia (6.3%), vomiting (5%), and elevated liver enzymes (1.3%). Fourteen patients (17.7%) were diagnosed via screening; 9 (64.3%) had type 1 diabetes. Endoscopy findings were consistent with CD in 77 patients; 2 had normal results. Non-celiac endoscopic findings were seen in 19 patients. Histopathology confirmed Marsh 3 lesions in 78 patients; 1 had normal findings, with tTG IgA levels normalizing at a 4-month follow-up. The positive predictive value of biopsy-free criteria was 98.7%. Non-celiac findings (in 24 patients) included *Helicobacter pylori* gastritis (n = 7) and eosinophilic esophagitis (n = 2).

**Conclusion:**

The biopsy-free diagnostic approach accurately identifies CD in most cases but may miss treatable conditions like eosinophilic esophagitis or *H*.* pylori* infection, especially in endemic regions. Misdiagnoses, though rare, highlight the need for careful evaluation in populations with diverse clinical presentations.

**Main Points**

- This study is the first to evaluate the applicability of ESPGHAN biopsy-free diagnostic criteria for celiac disease in a Turkish pediatric cohort.
- Although the biopsy-free approach had a high positive predictive value (98.7%), routine biopsies revealed additional non-celiac findings, such as *Helicobacter pylori* gastritis and eosinophilic esophagitis, emphasizing the value of endoscopy.
- The findings suggest that while ESPGHAN criteria are effective for most cases, their universal applicability in regions like Türkiye requires further validation due to unique healthcare and epidemiological factors.

## Introduction

Celiac disease (CD) is an autoimmune-mediated intestinal mucosal injury triggered by gluten ingestion in genetically predisposed individuals. It affects approximately 0.5%-2.5% of the global population.^1^ ^2^ In Türkiye, a large-scale study involving 20 190 students reported a biopsy-confirmed CD prevalence of around 1 in 212 individuals.^3^ Celiac disease can present with a wide range of clinical symptoms, including both intestinal and extraintestinal manifestations, or it may be detected in asymptomatic individuals through screening alone. Common to all patients, however, are the characteristic histopathological features observed in the intestine: intraepithelial lymphocytosis, crypt hyperplasia, and villous atrophy.^1^

In pediatric patients, until 2012, the diagnosis of CD was established through total IgA and anti-tissue transglutaminase IgA (tTG IgA) serologic screening, followed by endoscopic biopsy of the duodenum.^4^ However, in 2012, the European Society for Pediatric Gastroenterology, Hepatology and Nutrition (ESPGHAN) recommended a biopsy-free diagnostic approach for children with genetic susceptibility (positive Human Leukocyte Antigen) (HLA) typing), tTG IgA levels exceeding 10 times the upper normal limit, and a positive anti-endomysial antibody (EMA) test in a separate blood sample.^5^ In the latest ESPGHAN guidelines published in 2020, the requirement to confirm genetic susceptibility via HLA testing was removed, further simplifying the biopsy-free diagnostic process for patients with compatible serologic profiles.^6^

While biopsy-free diagnosis of CD has become widely adopted in European countries, it is not yet a routine practice in countries such as the United States, Australia, or Türkiye.^7^

In this study, the aim was to evaluate the endoscopic and histopathological findings, as well as the practical applicability of the new biopsy-free diagnostic criteria in patients who met ESPGHAN’s biopsy-free criteria but were nonetheless diagnosed through biopsy at the center.

## Materials and Methods

This study included pediatric patients under 18 years of age diagnosed with CD at the Eskişehir Osmangazi University Hospital over the past 5 years. For all patients referred to the clinic with a positive CD screening from other centers, or those suspected of having CD upon evaluation in the department, celiac screening tests were performed, including total serum immunoglobulin A (IgA) and tTG IgA; (provided by ORGENTEC, Mainz, Germany). For patients with low IgA levels, tTG IgG levels were measured. Patients with positive serologic screening for CD underwent endoscopy according to the ESPGHAN 2020 diagnostic algorithm.^6^ Among the screened patients, those with tTG-IgA levels exceeding 10 times the upper normal limit were evaluated according to the ESPGHAN 2020 biopsy-free diagnostic criteria for CD, which require confirmation with anti-EMA testing using the immunofluorescence method in a separate sample.

Patients who met the biopsy-free diagnostic criteria were informed about the option of a biopsy-free diagnosis; however, they were also told that biopsy-based diagnosis remains the preferred method in Türkiye. Biopsy samples were taken from the bulb and duodenum for patients who consented to the procedure. Six patients who met the biopsy-free criteria but opted not to undergo biopsy were started on a gluten-free diet without endoscopy. Ultimately, out of 180 patients diagnosed with CD through endoscopy due to positive serologic screening according to ESPGHAN 2020 criteria, 79 patients (43.8%) met the ESPGHAN criteria for biopsy-free diagnosis.

Routine endoscopic biopsy samples were obtained from all patients at diagnosis, including 4 from the duodenum, 2 from the bulb, and 2 each from the antrum, corpus, and esophagus. Demographic characteristics, clinical presentation, celiac serologic markers, endoscopic findings, and histopathological results were retrospectively analyzed.

Ethical approval was obtained from the Eskişehir Osmangazi University (approval date: February 27, 2024, decision no.: 68).

Statistical analyses were conducted using SPSS software version 24 (IBM SPSS Corp.;, Armonk, NY, USA). Quantitative data were presented as mean ± SD, and categorical variables as frequency and percentage. Comparisons of categorical data were performed using chi-square tests.

## Results

In this study, 79 patients (M/F: 38/41) met ESPGHAN criteria for biopsy-free diagnosis but underwent endoscopy. The mean age at diagnosis was 7.48 ± 4 years (range, 2-17 years).

Presenting symptoms included growth failure in 26 patients (32.9%), abdominal pain in 18 patients (22.8%), constipation in 7 patients (8.9%), diarrhea in 6 patients (7.6%), refractory/recurrent iron deficiency anemia in 5 patients (6.3%), vomiting in 4 patients (5%), and elevated liver enzymes in 1 patient (1.3%). Fourteen patients (17.7%) were diagnosed via screening: 9 of these patients (64.3%) had type 1 diabetes mellitus (T1DM), 4 (28.6%) had a first-degree relative with CD, and 1 (7.1%) had autoimmune thyroiditis. One patient with T1DM also had Down syndrome, while another had autoimmune thyroiditis.

Endoscopic examination revealed findings consistent with CD in 77 patients; however, 2 patients had a normal duodenal appearance. Non-celiac findings were noted in 19 patients (Table 1).

Histopathologically, all but 1 patient’s duodenal biopsies were consistent with Marsh-Oberhuber type 3 classification for CD: 13 patients (16.5%) were classified as type 3A, 49 (62%) as type 3B, and 16 (20.3%) as type 3C. The positive predictive value (PPV) of the ESPGHAN biopsy-free diagnostic criteria was calculated as 98.7%. One patient had a normal duodenal and bulb biopsy. This 4-year-old female patient had serum tTG IgA levels more than 10 times the upper limit of normal (ULN) and positive anti-EMA on 2 different occasions, which were tested twice at 2 different medical centers. It was noted that she had experienced recent acute gastroenteritis and had taken metronidazole prior to presentation. At her 4-month follow-up, CD serology (tTG IgA and EMA) had normalized, and she remained asymptomatic after 1 year.

In total, non-celiac histopathological findings were identified in 24 patients (30.4%) (Table 2). Of the 2 patients with eosinophilia in the esophagus, 1 was an 11.5-year-old male presenting with nausea, vomiting, and diarrhea. Although his esophagus appeared normal on endoscopy, a biopsy of the lower esophagus revealed 50 eosinophils per high-power field (HPF), with eosinophil degranulation and microabscesses. This patient did not attend follow-up visits. The second patient was a 3.5-year-old female referred for CD screening due to hypothyroidism. Her family history included allergic rhinitis in her father and asthma in her grandmother. Endoscopy showed pale mucosa with a “furrowing” pattern in the esophagus, and biopsies from the upper, middle, and lower esophagus revealed 40 eosinophils/HPF and eosinophilic microabscesses. She was started on milk elimination and a proton pump inhibitör, along with a gluten-free diet. A control biopsy after 4 months showed a normal endoscopic appearance but persistent eosinophilic infiltration (27 eosinophils/HPF).

Among 7 patients with histologically confirmed *Helicobacter pylori* infection, 3 had superficial ulcers in the bulb on endoscopy, and 5 received eradication therapy.

## Discussion

In 2020, ESPGHAN introduced simplified criteria for diagnosing CD without biopsy, removing the requirements for HLA typing and symptom presence.^6^ Currently, biopsy-free diagnosis relies on tTG IgA levels ≥10× ULN and positive EMA antibodies in a second sample. To our knowledge, this is the first study evaluating the applicability of biopsy-free celiac diagnosis in pediatric patients in Türkiye.

In studies conducted on celiac patients, the proportion of patients eligible for biopsy-free diagnosis according to the ESPGHAN 2020 criteria was reported to be 23% in Europe, whereas a study from North America found this rate to be 57%.^2^^,7^ In this study, it was determined that 79 out of 180 patients (43.8%) were eligible for biopsy-free diagnosis.

In this cohort, 17.7% (n = 14) of patients were asymptomatic and diagnosed through screening, meeting the biopsy-free criteria with biopsy results confirming CD. Although the sample size is limited, these findings suggest that ESPGHAN’s diagnostic algorithm could be effective in asymptomatic patients, aligning with prior studies.^8^^-^^10^ However, at this center, newly diagnosed asymptomatic T1DM patients undergo repeat testing after 6 months, with a biopsy recommended if CD markers remain positive, given the possibility of transient CD marker positivity in T1DM.^11^ This highlights an important point: biopsy-free diagnosis in asymptomatic patients, especially those newly diagnosed with T1DM, may require a more cautious approach.

While ESPGHAN’s no-biopsy CD criteria are increasingly used across Europe, their application remains limited in regions like North America, Australia, India, and Türkiye.^7^ ^12^ In societies with low-middle income, such as Türkiye, avoiding unnecessary CD diagnoses is essential, as a lifelong gluten-free diet significantly affects daily life and dietary practices.^13^ Furthermore, the cultural importance of high gluten consumption dietary habits in these regions underscores the need for precise, accurate diagnoses.^7^ ^14^

Our study demonstrated a high PPV of 98.7% for biopsy-free CD criteria, consistent with other research.^8^ ^15^ Nonetheless, it is noteworthy that 43.8% of the CD patients met the biopsy-free criteria. Additionally, with an estimated biopsy-proven CD prevalence of about 1 in 212 school children in Türkiye, the potential for false-positive diagnoses in the general population should not be ignored.^3^ Studies consistently highlight the need for reliable serologic testing for CD diagnosis.^7^ ^15^ ^16^ While European countries employ regular validation processes to ensure consistency across laboratories, such quality controls are often lacking in Türkiye.^17^ For instance, while anti-tTG IgA test cut-off values are well-defined in Europe, they remain unstandardized in the United States and Türkiye.^7^ Furthermore, most validation studies have predominantly focused on Caucasian populations, leaving limited data on other ethnic groups.^7^

Transient autoimmune positivity of CD markers has been reported in both adults and children.^18^ In 1 case from this cohort, a patient had anti-tTG IgA levels exceeding 10× ULN and positive EMA at 2 separate healthcare centers following acute gastroenteritis. However, both markers normalized within 4 months. Although uncommon, this case underscores the potential for misdiagnosis in patients with temporary autoimmune reactivity, particularly after infections such as gastroenteritis. Werkstetter et al^8^ reported that the PPV value for celiac markers in the non-biopsy diagnosis of CD is 100% when malabsorptive symptoms are present, which contrasts with this case. It is believed that caution is necessary when employing a biopsy-free approach in these scenarios.

The possibility of diagnosing CD in adults based solely on serological tests remains a subject of ongoing debate. The scientific community continues to discuss the potential benefits and drawbacks of implementing this approach.^19^ In adults with CD, about 30% experience persistent symptoms post diagnosis.^20^ Follow-up studies have shown that serologic markers do not reliably reflect mucosal healing, and initial endoscopic findings may provide useful insights for managing refractory cases and persistent symptoms in both children and adults.^7^ ^21^ Additionally, gastrointestinal malignancies, such as lymphoma, emphasize the importance of duodenal biopsy in adults, where anemia and potential malignancies in the CD differential diagnosis often necessitate endoscopic evaluation.^7^ Although malignancies are rare in children, endoscopy remains a useful tool for ruling out conditions like eosinophilic esophagitis or *H*. *pylori* infection, which might be missed in a biopsy-free approach.^22^ Guandalini and Newland^23^ reported in 2013 that up to 10% of pediatric patients eligible for biopsy-free CD diagnosis presented with non-CD-related findings on endoscopy. In this study, 30.4% of patients also had additional gastrointestinal findings, reinforcing the need for careful assessment.

The association between CD and eosinophilic esophagitis has been noted in previous studies.^24^ ^25^ Literature suggests that eosinophilic esophagitis risk may be increased in patients with CD, highlighting the need for further research into the frequency and reason for this association. In this cohort, eosinophilic esophagitis was found in 2.5% (n = 2) of patients, and in 1 case, esophageal eosinophilia persisted despite a gluten-free diet.

Similarly, a non-biopsy approach might overlook concurrent *H*. *pylori* infections, as symptoms can overlap, particularly in regions with high *H*. *pylori* prevalence.^26^ Furthermore, recent data from the EuroPedHp Registry showed that *H*. *pylori*-infected children with gastrointestinal comorbidities like CD, compared to those with no comorbidity showed a 75% reduced chance of receiving eradication therapy.^27^ In this cohort, *H*. *pylori* was identified in 7 patients, 5 of whom required treatment. The estimated *H*. *pylori* prevalence in Türkiye underscores the necessity of a more comprehensive approach to avoid missed diagnoses. In inflammatory bowel disease (IBD), particularly Crohn’s disease, upper gastrointestinal involvement can lead to mucosal atrophy and positive CD serology, with symptom relief upon adopting a gluten-free diet.^7^ In infants with ambiguous findings for CD or those with suspected very early-stage IBD, a biopsy-free celiac diagnosis should be approached with great caution.

In pediatric patients, endoscopy requires general anesthesia, posing risks like allergic reactions, respiratory complications, and rare procedural complications.^28^ The biopsy-free approach is less costly, particularly given the rarity of gastrointestinal cancers in children and the high sensitivity of current serologic tests, making it an appealing alternative. While duodenal biopsy is considered the gold standard for CD diagnosis, variability among pathologists and sample preparation errors can occasionally compromise diagnostic accuracy.^29^ Inadequate sampling is especially common in adults; studies have shown that two-thirds of adult patients in the United States had insufficient biopsy samples, contributing to both overdiagnosis and missed diagnoses.^30^ While these factors highlight the advantages of biopsy-free CD diagnosis, further evaluation with larger series is needed to determine the applicability of biopsy-free CD diagnosis in all patients in the country.

In conclusion, although biopsy-free CD diagnosis offers significant advantages, several limitations remain. These include the potential for missing concurrent conditions, variability in autoantibody levels due to lack of standardization among laboratories, and the risk of premature dietary interventions by healthcare providers outside gastroenterology. For these reasons, it is too early to implement universal biopsy-free CD diagnosis for all patients in Türkiye.

## Figures and Tables

**Table 1.** Endoscopic Findings of the Patients

	Number of Patients n = 79 (n, (%))
Endoscopic findings	
Findings consistent with CD	77 (97.5)
Normal findings	2 (2.5)
Findings other than duodenal mucosal atrophy	
Antral gastritis	13 (16.4)
Pangastritis	1 (1.3)
Eosinophilic esophagitis	1 (1.3)
Pancreatic rest	1 (1.3)
Antral polyp	1 (1.3)
Bezoar	1 (1.3)
Dilated lacteals in duodenum	1 (1.3)

CD, celiac disease.

**Table 2.** Histopathologic Findings Other Than Celiac Disease

Histopathologic Findings	N (%)
Gastric metaplasia in duodenum	12 (15.2)
*H*. *pylori* gastritis	7 (8.8)
Eosinophilic esophagitis	2 (2.5)
Lymphocytic gastritis	1 (1.3)
Hyperplastic polyp in the antrum	1 (1.3)
Intestinal metaplasia in the antrum	1 (1.3)

## Data Availability

The data that support the findings of this study are available on request from the corresponding author.
